# Budesonide/formoterol fumarate dihydrate *versus* budesonide with a novel suspension technology for uncontrolled asthma: phase 3 LITHOS study

**DOI:** 10.1183/23120541.01818-2025

**Published:** 2026-09-14

**Authors:** Alberto Papi, Robert A. Wise, David J. Jackson, Njira Lugogo, Ruchong Chen, Hassan Chami, Clara Armengol, Sami Z. Daoud, Emma Nauclér, Karin Bowen, Ayman Megally, Olami Sobande, Mehul Patel

**Affiliations:** 1 Respiratory Medicine, Department of Translational Medicine, University of Ferrara, Ferrara, Italy; 2 Department of Medicine, Johns Hopkins University School of Medicine, Baltimore, MD, USA; 3 Guy's Severe Asthma Centre, Guy's Hospital, School of Immunology and Microbial Sciences, King's College London, London, UK; 4 Division of Pulmonary and Critical Care Medicine, University of Michigan, Ann Arbor, MI, USA; 5 State Key Laboratory of Respiratory Disease, Joint International Research Laboratory of Respiratory Health, National Clinical Research Center for Respiratory Disease, National Center for Respiratory Medicine, Department of Allergy and Clinical Immunology, Guangzhou Institute of Respiratory Health, The First Affiliated Hospital of Guangzhou Medical University, Guangzhou, PR China; 6 Guangzhou National Laboratory, Guangzhou, PR China; 7 Respiratory and Immunology, Clinical Development, Biopharmaceuticals R&D, AstraZeneca, Gaithersburg, MD, USA; 8 Respiratory and Immunology, Clinical Development, Biopharmaceuticals R&D, AstraZeneca, Barcelona, Spain; 9 Respiratory and Immunology, Biometrics, Biopharmaceuticals R&D, AstraZeneca, Gothenburg, Sweden; 10 Respiratory and Immunology, Biometrics, Biopharmaceuticals R&D, AstraZeneca, Gaithersburg, MD, USA; 11 Respiratory and Immunology, Clinical Development, Biopharmaceuticals R&D, AstraZeneca, Cambridge, UK

## Abstract

**Background:**

Inhaled corticosteroid (ICS)/long-acting β_2_-agonist (LABA) therapy is a mainstay asthma treatment. LITHOS (clinicaltrials.gov identifier NCT05755906), a randomised phase 3 study, assessed efficacy and safety of low-dose budesonide/formoterol fumarate dihydrate (BFF) *via* a metered-dose inhaler (MDI) using Aerosphere co-suspension delivery technology (BFF_A_) *versus* low-dose budesonide *via* MDI using Aerosphere (BD).

**Methods:**

Participants (aged 12–80 years; N=374) with inadequately controlled asthma (seven-item Asthma Control Questionnaire score ≥1.5) despite low-dose ICS or ICS/LABA were randomised to 12 weeks of twice-daily BFF 160/10 μg (BFF_A_ 160) or BD 160 μg (BD 160). End-points included change from baseline in forced expiratory volume in 1 s (FEV_1_) area under the curve from 0–3 h (AUC_0–3_) (primary) and morning pre-dose trough FEV_1_ (key secondary) at week 12, onset of action, severe exacerbation rates and safety/tolerability.

**Results:**

Least-square mean differences (95% CI) demonstrated that BFF_A_ 160 provided superior lung function improvement *versus* BD 160 at week 12 for change from baseline in FEV_1_ AUC_0–3_ (200 (134–267) mL; p<0.0001) and morning pre-dose FEV_1_ (82 (20–144) mL; p=0.0096). Severe exacerbation rates were nominally reduced with BFF_A_ 160 *versus* BD 160 (rate ratio (95% CI) 0.48 (0.23–0.97); unadjusted p=0.0419). No new or unexpected safety findings were observed; on-treatment adverse effects were comparable between treatments (BFF_A_ 160, 18.4%; BD 160, 19.5%).

**Conclusion:**

These findings support the use of BFF_A_ 160 twice daily with novel Aerosphere technology for the treatment of people aged 12–80 years with asthma inadequately controlled by low-dose ICS or ICS/LABA.

## Shareable abstract

**Using Aerosphere co-suspension MDI technology, single-inhaler budesonide/formoterol was superior to budesonide on lung function, onset of action, rescue medication use and asthma control/QoL in people with inadequately controlled asthma** https://bit.ly/4dKmVfq

## Introduction

Asthma is a heterogeneous disease affecting adults and children that is characterised by chronic airway inflammation and defined by respiratory symptoms and variable levels of expiratory airflow limitation [1, 2]. The goals of asthma management include achieving symptom control, maintaining normal activity levels, and minimising the risk of asthma-related mortality, exacerbations, airflow limitation and adverse events associated with treatment [1]. Dual combinations of an inhaled corticosteroid (ICS) and a long-acting β_2_-agonist (LABA) provide an important treatment option for single maintenance and reliever (LABA with rapid onset of action) therapy in adults and adolescents with asthma [1, 3, 4]. The Global Initiative for Asthma recommendations for asthma maintenance therapy include a stepwise treatment approach in adults and adolescents aged ≥12 years; for people with moderate-to-severe asthma, this involves moving from low-dose ICS with bronchodilator therapy (preferred) or ICS alone to medium-dose or high-dose ICS/LABA maintenance therapy, with addition of a long-acting muscarinic antagonist (LAMA) considered for people whose asthma remains uncontrolled with ICS/LABA [1].

The dual ICS/LABA combination of budesonide and formoterol fumarate dihydrate in a standard suspension formulation (160/4.5 µg twice daily administered *via* a metered-dose inhaler (MDI)) was associated with greater improvements in lung function and lower exacerbation rates *versus* budesonide (BD) alone *via* MDI in studies of adolescents and adults with predominantly moderate-to-severe asthma [5, 6]. While ICS and ICS/LABA-containing maintenance therapies are the standard of care in asthma, a substantial proportion of people with asthma continue to experience poor control [1, 7, 8]. Moreover, the effectiveness of ICS/LABA combinations in MDI devices may be limited by adherence and use challenges, such as variable drug delivery [9]. These challenges demonstrate a need for additional options, including an ICS/LABA combination in a different inhaled format. Budesonide/formoterol MDI using Aerosphere co-suspension delivery technology is being developed for the treatment of asthma [10].

The co-suspension delivery technology consists of spray-dried porous particles with the phospholipid 1,2-distearoyl-sn-glycero-3-phosphocholine and calcium chloride suspended in a propellant [11, 12]. When used in combination MDIs, the particles form strong nonspecific associations with the active pharmaceutical agents, facilitating reproducible drug delivery and long-term stability [13]. This co-suspension technology is approved and available for use in >50 countries, including the United States of America (USA) and the European Union, as a single-inhaler budesonide/glycopyrronium/formoterol fumarate dihydrate triple therapy for maintenance treatment in people with COPD [10, 14, 15]. While approved dual ICS/LABA MDIs are important therapeutic options in asthma, an ICS/LABA Aerosphere co-suspension formulation represents another potential treatment option for people with asthma who do not achieve adequate disease control using currently available therapies.

Budesonide/formoterol fumarate dihydrate (BFF) delivered *via* an MDI using the same co-suspension delivery technology is being evaluated in two phase III clinical studies in asthma: LITHOS (clinicaltrials.gov identifier NCT05755906) and VATHOS (clinicaltrials.gov identifier NCT05202262). We report the results of LITHOS, a 12-week, phase 3 study that evaluated the impact of low-dose BFF *versus* BD, both using Aerosphere co-suspension delivery technology MDIs, on lung function and asthma health-related quality of life in participants aged 12–80 years with asthma who remain inadequately controlled despite low-dose ICS or ICS/LABA. The LITHOS study was designed to confirm the treatment benefits of combining formoterol fumarate dihydrate and budesonide in a novel co-suspension formulation compared with budesonide alone in individuals with inadequately controlled asthma.

## Methods

### Design and treatment

**FIGURE 1:**
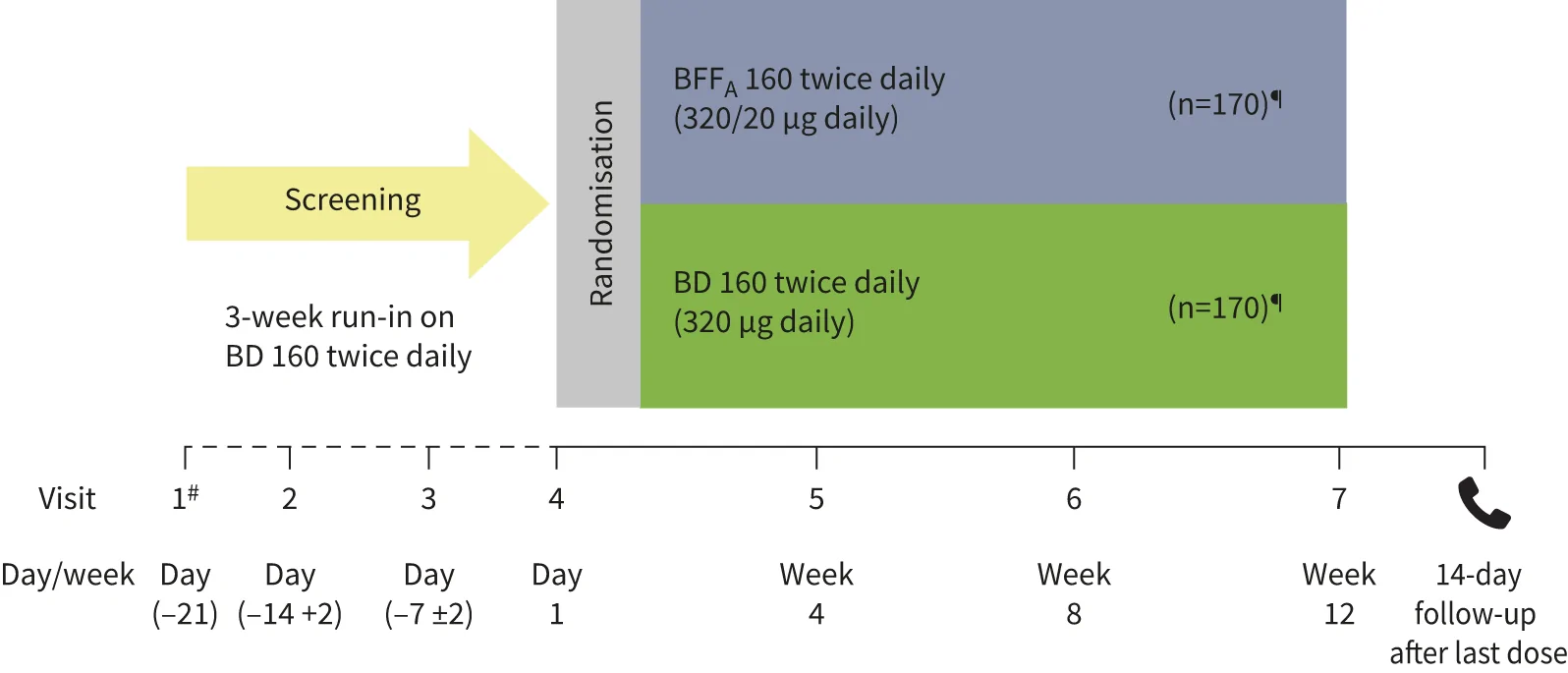
Study design schematic. BD 160: budesonide 160 μg *via* Aerosphere metered-dose inhaler (MDI); BFF_A_ 160: budesonide/formoterol fumarate dihydrate 160/10 μg *via* Aerosphere MDI. ^#^: stop daily inhaled corticosteroid (ICS)/long-acting β_2_-agonist or ICS regimen and start run-in with BD 160 twice daily; ^¶^: participant numbers (n=170) indicate the planned enrolment for each group.

LITHOS is a 12-week, phase 3, randomised, double-blind, parallel-group, multicentre study in individuals aged 12–80 years with inadequately controlled asthma (figure 1) and a forced expiratory volume in 1 s (FEV_1_) between 50% and 90% of predicted normal value. The study was conducted at 106 sites across seven countries (Canada, Czechia, Republic of Korea, Malaysia, Philippines, South Africa and USA) between 27 February 2023 and 19 November 2024. After meeting eligibility criteria, participants switched from their ICS or ICS/LABA regimen and initiated a run-in period with twice-daily BD MDI 160 µg (BD 160) at visit 1 until the evening before visit 4 (randomisation). The duration of the run-in/screening period was 3 weeks, and visit 4 occurred 21 days after visit 1. The run-in period provided time to stabilise the participants and ensure their safety before initiating the treatment phase. At visit 4, eligible participants discontinued the BD 160 run-in and were randomised 1:1 to BFF MDI 160/10 µg (BFF_A_ 160) or BD 160 twice daily. Participant randomisation was performed using an interactive response technology (IRT) system. The IRT system provided the study investigators or pharmacists with a kit identification number to be allocated to the participant at the dispensing visit in order to maintain the blinding between BFF MDI and BD MDI. Randomisation was stratified by baseline asthma treatment (ICS *versus* ICS/LABA) and age (12–<18 *versus* ≥18 years). Following randomisation, participants had three additional treatment visits over the 12-week treatment period. Participants' medication adherence was recorded in a daily eDiary that was evaluated at each visit during the study. An alert was generated when a participant was not consistently completing their eDiary and/or confirming on the eDiary that they had taken their study intervention. At the end of the treatment period, the participant's physician or investigator prescribed suitable alternative asthma therapy, not necessarily the original treatment. The study ended when the last randomised participant completed 12 weeks of randomised study treatment, and a 2-week safety follow-up phone call was conducted.

The study was conducted in accordance with the ethical principles of the Declaration of Helsinki and consistent with International Council for Harmonisation/good clinical practice, applicable regulatory requirements and study sponsor policies on bioethics. The study protocol and participant informed consent documents were reviewed and approved by local or country-specific controlling institutional review boards or independent ethics committees. Protocol details may be available upon request to the corresponding author. Participants, or their legally authorised representatives, were required to sign informed consent statements, with participants informed their participation was voluntary. As the study was conducted during the COVID-19 pandemic, study continuity measures were put into place to ensure pandemic-related disruptions were minimal.

### Population

Detailed inclusion and exclusion criteria are provided in supplementary table S1. In brief, eligible participants were required to meet the following criteria: aged 12–80 years (inclusive); documented history of physician-diagnosed asthma ≥6 months before visit 1; regularly using stable, daily low-dose ICS or an ICS/LABA for ≥8 weeks before visit 1; seven-item Asthma Control Questionnaire (ACQ-7) score ≥1.5 at visits 1 and 4; pre-bronchodilator/pre-dose FEV_1_ <90% pred at visits 1, 2 and 3, and pre-dose FEV_1_ 50–90% pred at visit 4 pre-randomisation; and demonstration of asthma stability during the run-in period. Reversibility to salbutamol, defined as a post-salbutamol increase in FEV_1_ of ≥12% and ≥200 mL in those aged ≥18 years or ≥12% only in those aged 12–<18 years, was also evaluated either during the 12 months before visit 1 or at visit 2 or visit 3. Participants receiving systemic corticosteroids, LAMAs, biologics, high-dose ICS/LABA and those with history of respiratory failure requiring mechanical ventilation were excluded.

### End-points

Due to regional differences in health authority requirements, different primary and secondary end-points were assessed in two separate regional approaches, namely USA and Europe/rest of world (EU/ROW). Data from all participants recruited across all geographies were used in the submission approaches, which differ by comparator, end-points and the multiple-testing approach used, according to the health authority responsible for reviewing the marketing authorisation, rather than recruitment location or nationality. For the USA regional approach, the primary and key secondary lung function end-points were change from baseline in FEV_1_ area under the curve over 0–3 h (AUC_0–3_) and change from baseline in morning pre-dose trough FEV_1_, respectively, for BFF_A_ 160 *versus* BD 160 at week 12. For the EU/ROW regional approach, the primary and key secondary lung function end-points were change from baseline in morning pre-dose trough FEV_1_ and change from baseline in FEV_1_ AUC_0–3_, respectively, for BFF_A_ 160 *versus* BD 160 over 12 weeks. Changes in lung function were pre-specified as primary and key secondary lung function end-points in both regional approaches. Additional secondary end-points included: onset of action, measured as change from baseline in FEV_1_ at 5 min post-dose on day 1 (both regional approaches); the rate of severe exacerbations (USA regional approach only); percentage of responders (≥0.5 point decrease) for the ACQ-7 and ACQ-5, and Asthma Quality of Life Questionnaire for age ≥12 years (AQLQ+12) at week 12 (USA regional approach) or over 12 weeks (EU/ROW regional approach); and change from baseline in the number of rescue medication inhalations per day over 12 weeks (both regional approaches). Safety assessments included the occurrence of adverse events and changes from baseline in vital signs and clinical laboratory parameters.

Here, we report the USA regional approach, with the EU/ROW regional approach findings included in the supplementary information.

### Statistical analyses

A sample size of 340 participants (170 per treatment) was planned to assess primary and key secondary end-points. This assumed that ∼15% of randomised participants would discontinue before week 12. For change from baseline in FEV_1_ AUC_0–3_, this sample size provided 99% power to detect a statistically significant difference (two-sided α=0.05) at week 12 (assuming a true difference±sd of 192±350 mL). For change from baseline in morning pre-dose trough FEV_1_, this sample size provided 80% power to detect a statistically significant difference (two-sided α=0.05) at week 12 (assuming a true difference±sd of 112±340 mL).

All comparisons were tested for superiority. Multiple testing procedures (MTPs) with type 1 error control for the analysis of the primary and secondary end-points were applied to meet regional registration requirements (supplementary figure S1). To account for multiplicity when testing the primary and secondary end-points, a combination of hierarchical testing and a Hochberg approach was used to control the overall type I error rate. p-values <0.05, but not statistically significant, either due to placement in the MTP or absence from the MTP, were noted as nominal.

Unless specified, all observed data were used for efficacy analyses, excluding participants who received new asthma medication in conjunction with discontinuation of the study medication (considered as an unfavourable response). Full details of the statistical analyses for all end-points can be found in the supplementary information. In brief, efficacy analyses were conducted in the efficacy set (all randomised participants who received any treatment). Change from baseline in FEV_1_ AUC_0–3_ and in morning pre-dose trough FEV_1_ were analysed using a repeated-measures analysis of covariance (ANCOVA) model. The model included treatment, visit, prior maintenance medication (ICS *versus* ICS-LABA) and treatment-by-visit interaction as categorical covariates, and baseline trough FEV_1_ and percent salbutamol reversibility as continuous covariates. Onset of action was assessed using a repeated-measures ANCOVA fitted to day 1 post-baseline data. Rates of severe asthma exacerbations were analysed using a negative binomial model. For patient-reported outcomes, the percentage of responders was assessed using a logistic regression model, while change from baseline in average daily rescue medication use was assessed using a repeated-measures ANCOVA model.

Safety analyses were conducted in the safety set (all randomised participants who received any treatment); data are reported descriptively, with no hypothesis testing performed.

## Results

### Participants

**FIGURE 2:**
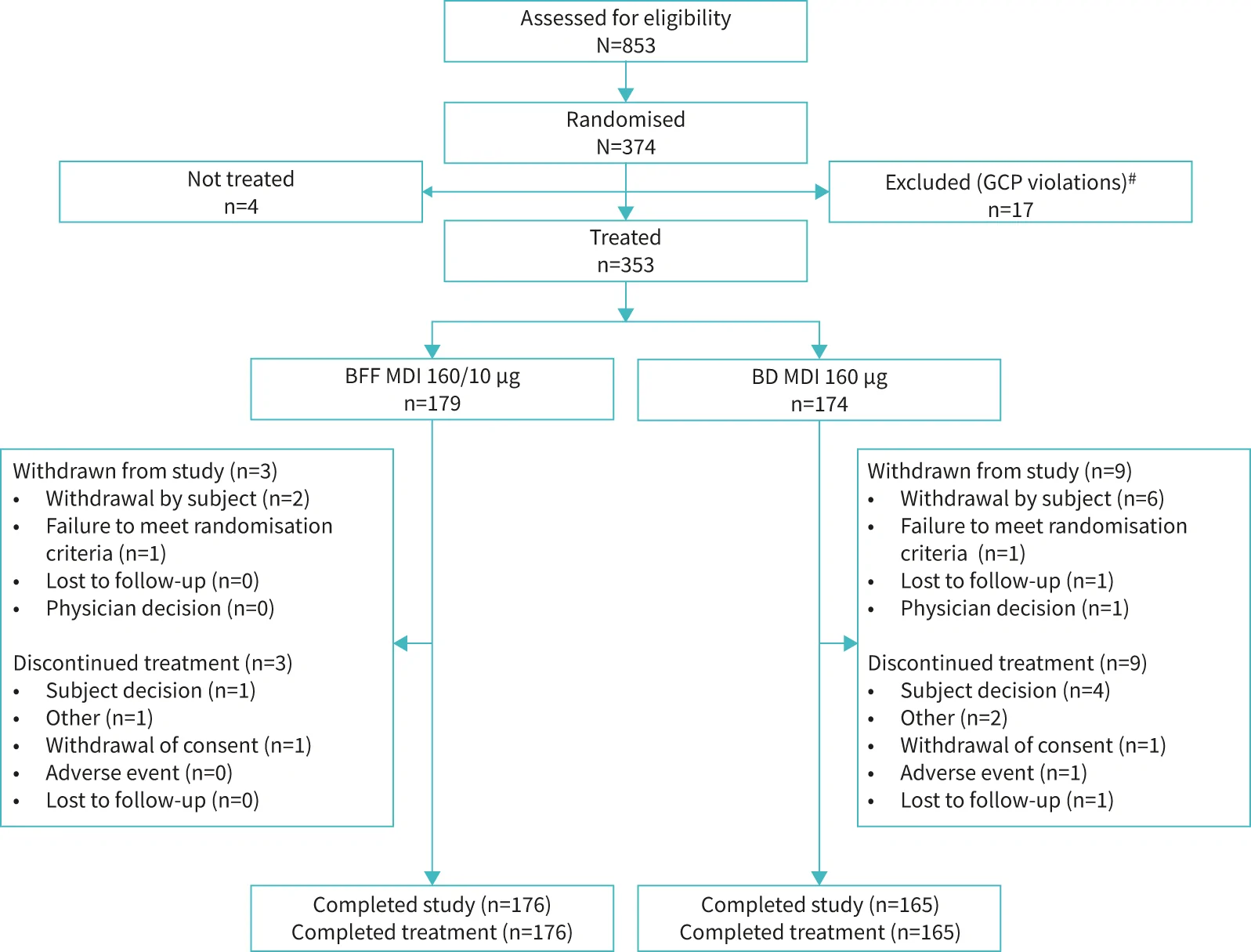
Participant disposition. Participants were randomised using a centralised interactive response technology (IRT) system. The participant, care provider, investigator and outcomes assessor were blinded during the study, with the IRT programmed with blind-breaking instructions, unless safety concerns or a regulatory requirement necessitated unblinding. GCP: good clinical practice; BFF_A_ 160: budesonide/formoterol fumarate dihydrate 160/10 μg *via* Aerosphere metered-dose inhaler (MDI); BD 160: budesonide 160 μg *via* Aerosphere MDI. ^#^: GCP violations were identified at four sites. Data from affected participants were excluded from all analysis sets except the screened set.

Participants were enrolled in the study between 27 February 2023 and 19 July 2024, and the last participant visit was 19 November 2024. Of a total 374 randomised participants, 353 were treated (BFF_A_ 160, n=179; BD 160, n=174) and 341 completed the study (BFF_A_ 160, n=176; BD 160, n=165; figure 2). 12 participants discontinued study medication (BFF_A_ 160, n=3; BD 160, n=9), with the most common reason for discontinuation being subject decision (n=5).

Participant demographics and baseline clinical characteristics are summarised in table 1. Baseline characteristics were generally well balanced across treatments, with most participants being female and aged between ≥18 and <65 years. The proportion of participants aged between ≥12 and <18 years was 3.2%. ∼75% of participants had been using stable daily ICS/LABA prior to the study.

**TABLE 1:** Demographics and clinical characteristics, efficacy set

	BFF_A_ 160	BD 160
**Subjects**	177	169
**Age years**	48.8±16.6	48.0±16.1
**Age group**		
≥12 to <18 years	6 (3.4)	5 (3.0)
≥18 to <65 years	139 (78.5)	130 (76.9)
≥65 to <75 years	28 (15.8)	29 (17.2)
≥75 years	4 (2.3)	5 (3.0)
**Male**	64 (36.2)	41 (24.3)
**Race**		
White	123 (69.5)	110 (65.1)
Asian	27 (15.3)	33 (19.5)
Black or African American	15 (8.5)	19 (11.2)
American Indian or Alaska Native	1 (0.6)	0
Native Hawaiian or other Pacific Islander	0	0
Multiple	2 (1.1)	2 (1.2)
Black or African American/other	2 (1.1)	1 (0.6)
White/other	0	1 (0.6)
Other	9 (5.1)	5 (3.0)
**Baseline percentage reversibility** ^#^	21.8±14.5	21.0±14.6
**Baseline blood eosinophil count cells·mm^−3^**	189.9±121.1	188.2±138.8
**Baseline blood eosinophil count cells·mm^−3^**		
<150	79 (44.6)	79 (46.7)
≥150 to <300	65 (36.7)	66 (39.1)
≥300	32 (18.1)	24 (14.2)
Missing	1 (0.6)	0
**Baseline pre-bronchodilator FEV_1_ mL**	2097±630	2047±607
**Prior asthma medication use** ^¶^		
ICS	43 (24.3)	42 (24.9)
ICS/LABA	134 (75.7)	127 (75.1)
**Baseline ACQ-7 score** ^+^	2.383±0.589	2.360±0.541
**Baseline severe asthma exacerbation history within the prior year**	0.14±0.380	0.17±0.408

Data are presented as n, mean±sd or n (%). BFF_A_ 160: budesonide/formoterol fumarate dihydrate 160/10 μg *via* Aerosphere metered dose inhaler (MDI); BD 160: budesonide 160 μg *via* Aerosphere MDI; FEV_1_: forced expiratory volume in 1 s; ICS: inhaled corticosteroids; LABA: long-acting β_2_-agonist; ACQ-7: seven-item Asthma Control Questionnaire. ^#^: reversibility was calculated as post-salbutamol FEV_1_ − pre-salbutamol FEV_1_. Reversibility (%) was calculated as (post-salbutamol FEV_1_ − pre-salbutamol FEV_1_) ÷ pre-salbutamol FEV_1_ × 100. Visit 2 values were used if nonmissing, otherwise visit 3 values were used. Reversibility at screening included only participants who were randomised based on reversibility at visit 2 or visit 3. ^¶^: baseline rescue medication was defined as the mean of nonmissing rescue medication values from the eDiary data collected during the last 7 days before randomisation. ^+^: defined as the last nonmissing ACQ value before randomisation.

### Primary and key secondary end-points

**FIGURE 3:**
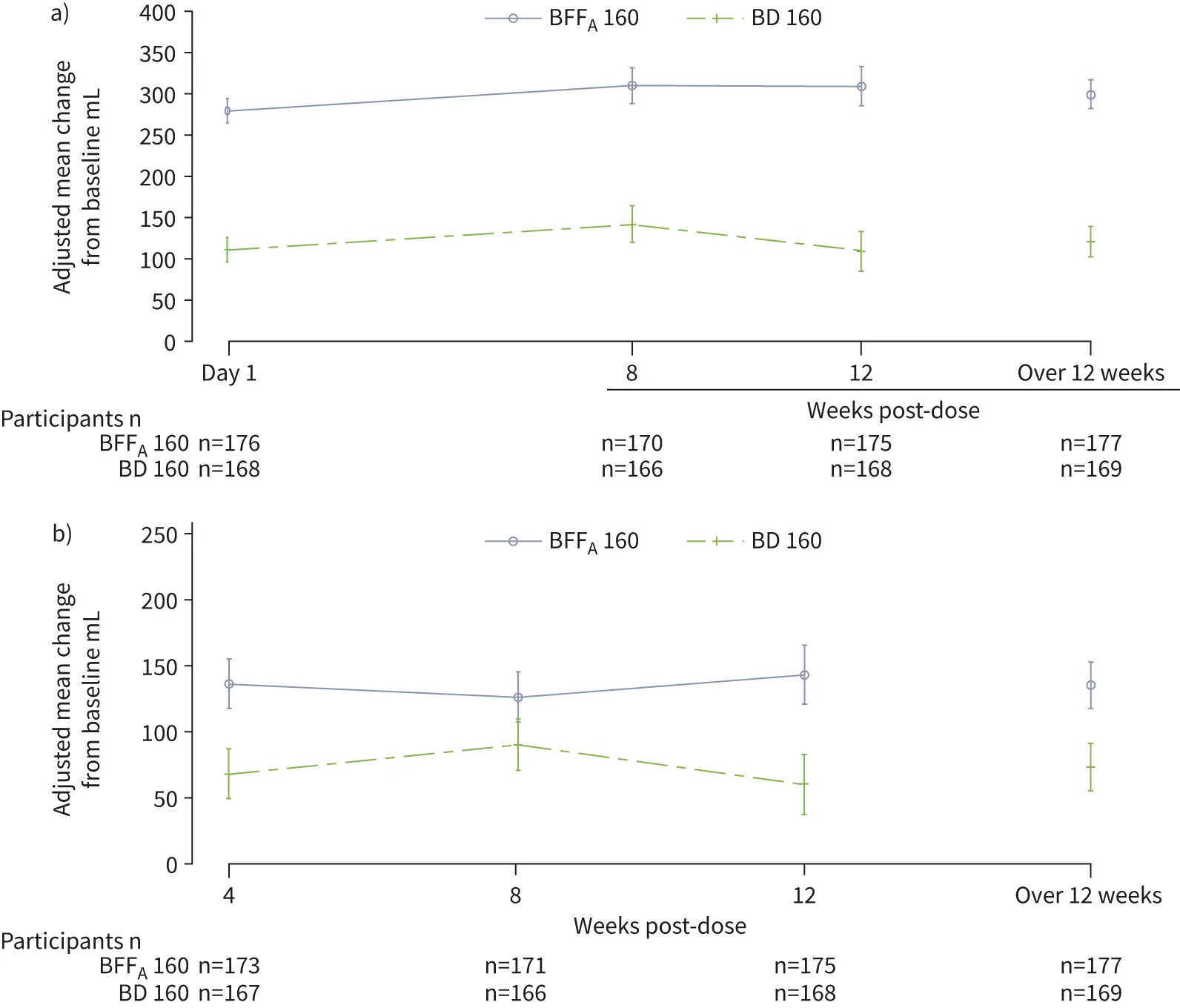
Change from baseline over time for a) forced expiratory volume in 1 s (FEV_1_) area under the curve over 0–3 h post-dose and b) morning pre-dose trough FEV_1_ (efficacy set) analysed using a repeated-measures analysis of covariance model. Further details on models and covariates used are presented in the supplementary material. BD 160: budesonide 160 μg *via* Aerosphere metered-dose inhaler (MDI); BFF_A_ 160: budesonide/formoterol fumarate dihydrate 160/10 μg *via* Aerosphere MDI.

At week 12, BFF_A_ 160 demonstrated statistically significantly greater change from baseline in FEV_1_ AUC_0–3_ compared with BD 160 (primary end-point; least squares mean (LSM) difference 200 mL, 95% CI 134–267 mL; p<0.0001; figure 3a; table 2) and in morning pre-dose trough FEV_1_ (key secondary end-point; LSM difference 82 mL, 95% CI 20–144 mL; p<0.0096; figure 3b; table 2).

**TABLE 2:** Summary of efficacy end-points, efficacy set^#^ (United States of America regional approach)

	BFF_A_ 160	BD 160	p-value
**Primary end-point**			
** **Change from baseline in FEV_1_ AUC_0–3_ at week 12^¶^ mL	n=175	n=168	
Estimate±se	309±23.5	109±24.1	
LS mean difference (95% CI)	200 (134–267)		<0.0001^¶¶^
**Secondary end-points included in the type I error control procedure and supportive end-points^+^**			
** **Key secondary end-point: change from baseline in morning pre-dose trough FEV_1_ at week 12^¶^ mL	n=175	n=168	
Estimate±se	143±22.1	60±22.6	
LS mean difference (95% CI)	82 (20–144)		<0.0096^¶¶^
Onset of action: change in FEV_1_ at 5 min post-dose on day 1^§^ mL	n=168	n=157	
Estimate±se	179±13.8	26±14.2	
LS mean difference (95% CI)	153 (114–192)		<0.0001^¶¶^
Percentage of ACQ-7 responders at week 12^ƒ^	n=175	n=168	
Responders, n (%)	130 (74.3)	104 (61.9)	
OR (95% CI), p-value	1.851 (1.158–2.958)		0.0101^¶¶^
LS mean difference in change from baseline (95% CI)^¶,+^	−0.251 (−0.402–−0.100)		0.0012^++^
Percentage of ACQ-5 responders at week 12^§^	n=175	n=168	
Responders, n (%)	135 (77.1)	105 (62.5)	
OR (95% CI)	2.114 (1.302–3.432)		0.0025^¶¶^
LS mean difference in change from baseline (95% CI)^¶,+^	−0.280 (−0.463–−0.097)		0.0028^++^
Percentage of AQLQ(s)+12 responders at week 12^ƒ^	n=172	n=162	
Responders, n (%)	106 (61.6)	77 (47.5)	
OR (95% CI)	1.873 (1.175–2.985)		0.0084^¶¶^
LS mean difference in change from baseline (95% CI)^¶,+^	0.265 (0.088–0.442)		0.0035^++^
Change from baseline in mean rescue medication use over 12 weeks, inhalations per day^##^	n=171	n=167	
Estimate±se	−0.589±0.0708	−0.309±0.0721	
LS mean difference (95% CI)	−0.280 (−0.478– −0.082)		0.0058^¶¶^

BFF_A_ 160: budesonide/formoterol fumarate dihydrate 160/10 μg *via* Aerosphere metered-dose inhaler (MDI); BD 160: budesonide 160 μg *via* Aerosphere MDI; FEV_1_: forced expiratory volume in 1 s; AUC_0–3_: area under the curve over 0–3 h post-dose; LS: least squares; ACQ-7/5: seven-/five-item Asthma Control Questionnaire; AQLQ(s)+12: (standardised) Asthma Quality of Life Questionnaire for age ≥12 years. ^#^: all observed data were used in analyses, unless participants discontinued prematurely in conjunction with asthma therapy or prohibited medication potentially impacting efficacy, in which case treatment failure was imputed; ^¶^: analysed using a repeated-measures analysis of covariance (ANCOVA) model; the model included treatment, visit, prior maintenance medication (inhaled corticosteroids (ICS) *versus* ICS/long-acting β_2_-agonist (LABA)) and treatment-by-visit interaction as categorical covariates, and the baseline trough FEV_1_ and percentage reversibility as continuous covariates. Further details on models and covariates used are presented in the supplementary material; ^+^: change from baseline (LS mean difference) in ACQ-5 and ACQ-7, and AQLQ were assessed as part of pre-specified analyses and were not part of the multiple testing procedure; ^§^: assessed using a repeated-measures ANCOVA fitted to day 1 post-baseline data; ^ƒ^: assessed using logistic regression; ^##^: assessed using a repeated-measures ANCOVA model. The model included treatment, the number of the relevant 4-week interval (interval 1 to 3), prior maintenance medication (ICS *versus* ICS-LABA), severe asthma exacerbation history (0, ≥1) and the treatment-by-4-week interval interaction as categorical covariates, and baseline daily rescue salbutamol use, baseline trough FEV_1_, and percentage salbutamol reversibility as continuous covariates; ^¶¶^: significant per the type I error control procedure; ^++^: unadjusted.

Treatment effects consistently favoured BFF_A_ 160 *versus* BD 160 across subgroups for end-points of change from baseline in FEV_1_ AUC_0–3_ or change from baseline in morning pre-dose trough FEV_1_ at week 12 (supplementary figure S2). There were too few participants for clinically meaningful analysis in adolescents and some racial subgroups.

Consistent findings were observed for primary and key secondary lung function end-points over 12 weeks using the EU/ROW regional approach (supplementary table S2).

### Secondary end-points

For onset of action, change from baseline in FEV_1_ at 5 min post-dose on day 1 demonstrated statistically significant improvement with BFF_A_ 160 *versus* BD 160 (table 2; supplementary figure S3).

The percentages of ACQ-7 and ACQ-5 responders (≥0.5 score decrease from baseline) and AQLQ+12 responders (≥0.5 score increase from baseline) were significantly greater with BFF_A_ 160 *versus* BD 160 at week 12 (table 2). In addition, the tertiary/exploratory end-point of treatment differences in change from baseline in the ACQ-7, ACQ-5 and AQLQ were not subject to type I error control and were considered nominally significant (table 2). Additionally, *post hoc* analyses showed that the percentage of participants with ACQ-7 scores <1.5 at week 12 was 62.3% (109 out of 175) with BFF_A_ 160 *versus* 45.8% (77 out of 168) with BD 160 (OR 2.348, 95% CI 1.463–3.769). There was a larger decrease from baseline in ACQ-5 scores at 4, 8 and 12 weeks, and over 12 weeks with BFF_A_ 160 *versus* BD 160 (supplementary figure S4).

Significantly greater reductions from baseline in mean daily rescue medication inhalations were observed for BFF_A_ 160 *versus* BD 160 (table 2). The tertiary/exploratory end-point of percentage of symptom-free days over 12 weeks while on treatment was higher with BFF_A_ *versus* BD 160 and was nominally significant due to absence of type I error control (supplementary table S3).

Generally, comparable findings for asthma control, health-related quality of life and rescue medication use were observed over 12 weeks for the EU/ROW regional approach (supplementary table S2), although the ACQ and AQLQ+12 responder analyses were only numerically greater with BFF_A_ 160 *versus* BD 160.

The rate ratio (95% CI) for severe exacerbations was nominally lower for BFF_A_ 160 *versus* BD 160 but was not statistically significant due to the type I error control procedure (0.48, 95% CI 0.23–0.97; p=0.0419; table 3). The overall number of participants experiencing exacerbations and the number of total exacerbations during the study was low (table 3).

**TABLE 3:** Rate of severe exacerbations, efficacy set

	BFF_A_ 160	BD 160
**Participants**	177	169
**Participants experiencing exacerbations**	12 (6.8)	21 (12.4)
**Exacerbations**	12	22
**Total follow-up time years**	41.1	38.6
**Adjusted annual exacerbation rate^#^** **±se**	0.25±0.08	0.52±0.12
**Rate ratio^#^** **(95% CI), p-value**	0.48 (0.23–0.97), p=0.0419^¶^	

Data are presented as n or n (%), unless otherwise stated. BFF_A_ 160: budesonide/formoterol fumarate dihydrate 160/10 μg *via* Aerosphere metered-dose inhaler (MDI); BD 160: budesonide 160 μg *via* Aerosphere MDI. ^#^: analysed using a negative binomial regression model including treatment, baseline trough forced expiratory volume in 1 s, percentage salbutamol reversibility, baseline severe asthma exacerbation history (0, ≥1), prior maintenance medication (inhaled corticosteroid (ICS) *versus* ICS/long-acting β_2_-agonist) and region as covariates. The logarithmic time at risk of experiencing a severe exacerbation used as an offset variable. ^¶^: nominally significant, but not statistically significant due to the type I error control procedure.

### Safety

Between BFF_A_ 160 and BD 160, no substantive differences were observed in the occurrence of on-treatment adverse events (BFF_A_ 160, 18.4%; BD 160, 19.5%) serious adverse events (SAEs) or adverse events leading to discontinuation (table 4). The most frequently reported adverse event in both treatment groups was upper respiratory tract infection. No on-treatment SAEs, including on-treatment SAEs with the outcome of death, were reported in either treatment group, and no paradoxical bronchospasms were reported during the study. In addition, no clinically meaningful trends were observed in clinical laboratory parameters, vital signs and physical findings over time, with values comparable between treatments.

**TABLE 4:** Summary of overall adverse events and most frequently reported adverse events, safety set

	BFF_A_ 160	BD 160
**Participants**	179	174
**Overall adverse events**		
Any adverse event	33 (18.4)	34 (19.5)
Any SAE	0 (0)	0 (0)
Any SAE with outcome of death	0 (0)	0 (0)
Any adverse event leading to discontinuation of study drug	0 (0)	1 (0.6)
**Most frequently reported adverse events** ^#^		
Upper respiratory tract infection	6 (3.4)	5 (2.9)
Nasopharyngitis	3 (1.7)	4 (2.3)

Data are presented as n or n (%). The table includes adverse events with an onset date on or after the date of first dose of study drug up to and including 1 day following the date of last study drug dose. Subjects with multiple occurrences in the same category were counted once per category regardless of the number of occurrences. BFF_A_ 160: budesonide/formoterol fumarate dihydrate 160/10 μg *via* Aerosphere metered-dose inhaler (MDI); BD 160: budesonide 160 μg *via* MDI; SAE: serious adverse event. ^#^: occurring in >2% of participants, by preferred term based on Medical Dictionary for Regulatory Activities (version 27.1).

## Discussion

This phase 3, randomised, double-blind 12-week study demonstrated that single-inhaler BFF delivered with an MDI utilising Aerosphere co-suspension technology (BFF_A_ 160) was superior to a BD MDI utilising Aerosphere co-suspension technology (BD 160) in improving multiple lung function end-points and use of rescue medication in participants aged 12–80 years with inadequately controlled asthma despite ICS or ICS/LABA treatment. The superiority of BFF_A_ 160 *versus* BD 160 on lung function improvement was demonstrated on primary and key secondary lung function end-points for both USA and EU/ROW regional approaches, including post-dose FEV_1_ AUC_0–3_ and pre-dose trough FEV_1_.

These findings were further supported in pre-defined subgroup analyses of the primary end-points, which showed consistent effects favouring BFF_A_ 160 across subgroups, including participants with inadequate asthma control despite prior dual ICS/LABA treatment. In addition, BFF_A_ 160 demonstrated a significantly faster onset of action *versus* BD 160. These findings are consistent with multiple studies demonstrating greater improvements in lung function with budesonide/formoterol fumarate dihydrate MDI *versus* budesonide MDI in a standard suspension formulation in individuals with asthma [6, 16, 17].

Because post-dose FEV_1_ AUC_0–3_ (the primary end-point in the USA analysis approach) and pre-dose trough FEV_1_ (the primary end-point in the EU/ROW approach) may reflect short-term bronchodilator and pharmacodynamic responses rather than indicating long-term disease control, additional outcomes were measured. These outcomes demonstrated that BFF_A_ improved asthma control and health-related quality of life, while reducing exacerbation rates *versus* BD 160. Moreover, BFF_A_ was well tolerated.

Significantly greater reductions in rescue medication use were observed across regional approaches with BFF_A_ 160 *versus* BD 160. Furthermore, significantly greater percentages of responders were observed with BFF_A_ 160 on the ACQ-5, ACQ-7 and AQLQ+12 at week 12, with numerically greater percentages of responders observed over 12 weeks. These data indicate that participants receiving BFF_A_ 160 were less bothered by asthma symptoms and experienced less impairment of their quality of life than those receiving BD 160 alone. Previous studies have reported greater asthma symptom control and health-related quality of life benefits, as well as greater treatment satisfaction, with BFF MDI *versus* BD MDI administered in the original standard suspension formulations [5, 6, 17, 18]. Data from real-world studies show that the high symptom burden associated with uncontrolled asthma impacts patient health status and productivity, including absenteeism [19, 20], highlighting the relevance of our findings for people aged 12–80 years with uncontrolled asthma.

The rate of severe exacerbations was a secondary outcome. The 12–week LITHOS study was primarily designed to assess lung function and was not powered to evaluate severe exacerbations. During the 12-week study, yielding ∼40 patient-years of follow-up per treatment group, the overall rates of severe exacerbations in the BFF_A_ 160 and BD 160 groups were relatively low and nominally lower in BFF_A_ 160 *versus* BD 160. While this did not reach statistical significance with the type I error hierarchical testing design, it is an important and clinically relevant outcome to report. The phase 3 VATHOS (NCT05202262) study of BFF MDI with co-suspension delivery technology has a 24-week study duration and includes a pre-specified analysis of pooled exacerbation data from VATHOS and LITHOS, which are reported in a separate co-published article [21].

Over the 12 weeks of the study, there were no new or unexpected tolerability or safety findings with BFF_A_ 160, no increased incidence of adverse events and no paradoxical bronchospasms *versus* BD 160. In addition, no SAEs, including on-treatment SAEs with the outcome of death, were reported during the study, and no clinically meaningful trends were observed for clinical laboratory parameters, vital signs or physical findings with BFF_A_ 160 *versus* BD 160. A favourable safety profile for BFF_A_ 160 was previously reported in the TELOS study in participants with COPD [22] and is consistent with the widely established safety profile of other BFF inhaled formulations in people with asthma or COPD [23–25].

The BFF study medication used in LITHOS contains the same molecules delivered by MDI or by dry-powder inhaler in Symbicort, which is approved in asthma and COPD indications [23–25]. However, here BFF was delivered using Aerosphere co-suspension delivery technology which provides optimised drug delivery, owing to low-density porous phospholipid particles [13].

Limitations of this study include the short 12-week study duration. This timeline is generally considered adequate for assessing lung function and was agreed upon by regulatory authorities; however, this, along with the participant selection criteria, resulted in a low number of exacerbation events, thus limiting the ability to assess the effect of treatment on exacerbations. In addition, there were few adolescent study participants aged <18 years for subgroup analysis, which may limit the generalisability of these results to the adolescent population with uncontrolled asthma. Finally, although lung function end-points in asthma do not always correlate with durable long-term responses, budesonide and formoterol have a long history of use in asthma, and we expect long-term outcomes to be similar to those previously observed with these agents.

In conclusion, these findings support the use of BFF_A_ 160, an MDI incorporating a novel co-suspension technology (Aerosphere) for the treatment of people aged 12–80 years with inadequately controlled asthma, demonstrating improved lung function, asthma control, health-related quality of life and reduced use of rescue medication with BFF_A_ 160 *versus* BD 160. The data reported here are consistent with other LABAs, and the role of ICS/LABA dual combination therapy is supported by physicians and the Global Initiative for Asthma as the accepted standard of care [1].

## Data Availability

Data underlying the findings described in this manuscript may be obtained in accordance with AstraZeneca's data sharing policy described at https://astrazenecagrouptrials.pharmacm.com/ST/Submission/Disclosure. Data for studies directly listed on Vivli can be requested through Vivli at https://www.vivli.org. Data for studies not listed on Vivli could be requested through Vivli at https://vivli.org/members/enquiries-about-studies-not-listed-on-the-vivli-platform/. The AstraZeneca Vivli member page is also available outlining further details: https://vivli.org/ourmember/astrazeneca/.

## Supplementary material

**Please note:** supplementary material is not edited by the Editorial Office, and is uploaded as it has been supplied by the author. Supplementary material 01818-2025.SUPPLEMENT

## References

[1] Global Initiative for Asthma. 2025 GINA Report: Global Strategy for Asthma Management and Prevention 2025. Date last accessed: 9 July 2025. https://ginasthma.org/2025-gina-strategy-report/

[2] Papi A, Hughes R, Del Olmo R, et al. Relationships between symptoms and lung function in asthma and/or chronic obstructive pulmonary disease in a real-life setting: the NOVEL observational longiTudinal studY. Ther Adv Respir Dis 2024; 18: 17534666241254212. doi:10.1177/17534666241254212 38841799 PMC11155362

[3] Reddel HK, Bateman ED, Schatz M, et al. A practical guide to implementing SMART in asthma management. J Allergy Clin Immunol Pract 2022; 10: S31–S38. doi:10.1016/j.jaip.2021.10.011 34666208

[4] Krings JG, Beasley R. The role of ICS-containing rescue therapy *versus* SABA alone in asthma management today. J Allergy Clin Immunol Pract 2024; 12: 870–879. doi:10.1016/j.jaip.2024.01.011 38237858 PMC10999356

[5] Peters S, Bleecker E, Canonica GW, et al. Serious asthma events with budesonide plus formoterol *vs*. budesonide alone. N Engl J Med 2016; 375: 850–860. doi:10.1056/NEJMoa1511190 27579635

[6] Noonan M, Rosenwasser LJ, Martin P, et al. Efficacy and safety of budesonide and formoterol in one pressurised metered-dose inhaler in adults and adolescents with moderate to severe asthma: a randomised clinical trial. Drugs 2006; 66: 2235–2254. doi:10.2165/00003495-200666170-00006 17137405

[7] Bae E, Park HJ, Park H, et al. Early clinical remission and its role in lung function decline and exacerbation in adult Korean patients with asthma. Thorax 2025; 80: 273–282. doi:10.1136/thorax-2024-222679 40050022

[8] Davis J, Trudo F, Siddall J, et al. Burden of asthma among patients adherent to ICS/LABA: a real-world study. J Asthma 2019; 56: 332–340. doi:10.1080/02770903.2018.1455858 29624458

[9] Usmani OS, Lavorini F, Marshall J, et al. Critical inhaler errors in asthma and COPD: a systematic review of impact on health outcomes. Respir Res 2018; 19: 10. doi:10.1186/s12931-017-0710-y 29338792 PMC5771074

[10] AstraZeneca. AstraZeneca announces the completion of the clinical programme to support the transition of Breztri to next-generation propellant with near-zero global warming potential. 2024. Date last accessed: 9 September 2024. www.astrazeneca.com/media-centre/press-releases/2024/astrazeneca-announces-the-completion-of-the-clinical-programme-to-support-the-transition-of-breztri-to-next-generation-propellant-with-near-zero-global-warming-potential.html

[11] Lechuga-Ballesteros D, Noga B, Vehring R, et al. Novel cosuspension metered-dose inhalers for the combination therapy of chronic obstructive pulmonary disease and asthma. Future Med Chem 2011; 3: 1703–1718. doi:10.4155/fmc.11.133 21942257

[12] Vehring R, Lechuga-Ballesteros D, Joshi V, et al. Cosuspensions of microcrystals and engineered microparticles for uniform and efficient delivery of respiratory therapeutics from pressurized metered dose inhalers. Langmuir 2012; 28: 15015–15023. doi:10.1021/la302281n 22985189

[13] Singh D, Roche N, Wu L, et al. *In silico* lung deposition profiles of three single-inhaler triple therapies in patients with COPD using functional respiratory imaging. Int J Chron Obstruct Pulmon Dis 2025; 20: 2103–2116. doi:10.2147/COPD.S510214 40606813 PMC12214429

[14] US Food and Drug Administration. Breztri Aerosphere™ Highlights of Prescribing Information. 2022. Date last accessed: 9 July 2025. www.accessdata.fda.gov/drugsatfda_docs/label/2020/212122s000lbl.pdf

[15] European Medicines Agency. Trixeo Aerosphere™ Summary of Product Characteristics. 2020. Date last accessed: 9 July 2025. www.ema.europa.eu/en/documents/product-information/trixeo-aerosphere-epar-product-information_en.pdf

[16] Corren J, Korenblat PE, Miller CJ, et al. Twelve-week, randomized, placebo-controlled, multicenter study of the efficacy and tolerability of budesonide and formoterol in one metered-dose inhaler compared with budesonide alone and formoterol alone in adolescents and adults with asthma. Clin Ther 2007; 29: 823–843. doi:10.1016/j.clinthera.2007.05.011 17697902

[17] Kerwin EM, Oppenheimer JJ, LaForce C, et al. Efficacy and tolerability of once-daily budesonide/formoterol pressurized metered-dose inhaler in adults and adolescents with asthma previously stable with twice-daily budesonide/formoterol dosing. Ann Allergy Asthma Immunol 2009; 103: 62–72. doi:10.1016/S1081-1206(10)60145-7 19663129

[18] Chervinsky P, Baker J, Bensch G, et al. Patient-reported outcomes in adults with moderate to severe asthma after use of budesonide and formoterol administered *via* 1 pressurized metered-dose inhaler. Ann Allergy Asthma Immunol 2008; 101: 463–473. doi:10.1016/S1081-1206(10)60284-0 19055199

[19] Ding B, Chen S, Srivastava D, et al. Symptom burden, health status, and productivity in patients with uncontrolled and controlled severe asthma in NOVELTY. J Asthma Allergy 2023; 16: 611–624. doi:10.2147/JAA.S401445 37334017 PMC10274410

[20] Lee LK, Ramakrishnan K, Safioti G, et al. Asthma control is associated with economic outcomes, work productivity and health-related quality of life in patients with asthma. BMJ Open Respir Res 2020; 7: e000534. 10.1136/bmjresp-2019-000534 PMC7101043 32193226

[21] Jackson DJ, Wise RA, Papi A, et al. Budesonide/formoterol fumarate dihydrate *versus* budesonide with a novel suspension technology for uncontrolled asthma: phase 3 VATHOS study. ERJ Open Res 2026; 12: 01817–2025. [ 10.1183/23120541.01817-2025].

[22] Ferguson GT, Papi A, Anzueto A, et al. Budesonide/formoterol MDI with co-suspension delivery technology in COPD: the TELOS study. Eur Respir J 2018; 52: 1801334. doi:10.1183/13993003.01334-2018 30220648 PMC6383599

[23] Electronic Medicines Compendium. Symbicort® Turbohaler® 200/6 Inhalation Powder: Summary of Product Characteristics. 2018. Date last accessed: 1 December 2025. www.medicines.org.uk/emc/medicine/4821

[24] AstraZeneca Pharmaceuticals. Symbicort Prescribing Information. 2017. Date last accessed: 1 December 2025. www.azpicentral.com/symbicort/symbicort.pdf

[25] AstraZeneca Pharmaceuticals LP. Symbicort Summary of Product Characteristics. 2017. Date last accessed: 1 December 2025. www.medicines.org.uk/emc/medicine/32036

