# Supplementary material for: Budesonide/formoterol fumarate dihydrate versus budesonide with a novel suspension technology for uncontrolled asthma: phase 3 LITHOS study
Source: ERJ Open Res. 2026 Sep 14;12(5):01818-2025. doi: 10.1183/23120541.01818-2025 (PMC13573226; doi:10.1183/23120541.01818-2025)
Supplement: Supplementary file 1 [file 01818-2025.SUPPLEMENT.pdf]

SUPPLEMENTARY INFORMATION

Table of contents

***Supplementary Appendix* 2**

Protocol amendments 2

Sample size (Europe/ROW)..2

Statistical analyses 3

Supplementary Table S1. Summary of key inclusion and exclusion criteria............................ 5

Supplementary Table S2. Summary of treatment differences for efficacy endpoints, (Europe/ROW regional approach), Efficacy Set. 7

Supplementary Figure S1. Overview of Type I error control MTPs for A) US regional approach; B) EU/ROW regional approach. 10

Supplementary Figure S2. Subgroup analyses for primary endpoints of change from baseline in FEV_1 $AUC_{0–3}$ at Week 12 by US regional approach (A) and in morning pre-dose trough FEV_1 over 12 weeks by EU/ROW regional approach (B). 12

Supplementary Figure S3. Change from baseline in onset of action on Day 1, Efficacy Set... 15

Supplementary Figure S4. Change from baseline in ACQ-5 by timepoint............................. 16

Supplementary Appendix

Protocol amendments

Amendments to the protocol were made after initiating participant recruitment. Severe asthma exacerbation rate analyses (pooled and individual studies) were added after feedback from a health authority. The onset of action assessment was changed to the difference in mean change from baseline and was no longer performed as a within group test but compared BFF_A 160 with BD 160 in the multiple testing procedure; the within-group test was retained as a supportive/exploratory analysis. The Type I error control procedure for the US regional approach was updated to use the Treatment Policy estimand and a subsequent amendment further updated the strategies for handling intercurrent events to reflect a composite strategy (treatment failure) if new asthma therapy or administration of prohibited medication potentially impacting efficacy occurs in conjunction with premature discontinuation per feedback from a Health Authority; otherwise, Treatment Policy strategy was used. Type I error control procedure for EU/ROW was updated to reflect onset of action being tested separately, outside the Hochberg test procedure.

Sample size (Europe/ROW)

A sample size of 340 participants (170 per treatment) was planned to assess primary and key secondary endpoints. For change from baseline in FEV_1 $AUC_{0–3}$, this sample size provided >99% power to detect a statistically significant difference (two-sided alpha 0.05) over 12 weeks (assuming a true difference of 200 mL [SD, 308 mL]). For change from baseline in morning pre-dose trough FEV_1, this sample size provided 92% power to detect a statistically significant difference (two-sided alpha 0.05) over 12 weeks (assuming a true difference of 120 mL [SD, 300 mL]).

Statistical analyses

Change from baseline in FEV_1 $AUC_{0–3}$ and in morning pre-dose trough FEV_1 were analysed using a repeated measures analysis of covariance (ANCOVA) model. The model included treatment, visit, prior maintenance medication (ICS *versus* ICS/LABA) and treatment-by-visit interaction as categorical covariates, and baseline trough FEV_1 and percent salbutamol reversibility as continuous covariates. Data following initiation of new asthma therapy in conjunction with premature study treatment discontinuation were imputed with the minimum FEV_1 value of 12% decrease from the participant's baseline or the participant's worst observed value post baseline. The rate of severe asthma exacerbations was analysed using a negative binomial model including treatment, baseline trough FEV_1, percent salbutamol reversibility, baseline severe asthma exacerbation history (0, ≥1), prior maintenance medication (ICS *versus* ICS/LABA) and region as covariates. The logarithmic time at risk of experiencing a severe exacerbation was used as an offset variable in the model. Percentage of responders on 7-item ACQ, 5-item ACQ and AQLQ+12 (defined as ≥0.5 score decreases) was assessed using logistic regression with treatment and prior maintenance medication (ICS *versus* ICS/LABA) as categorical covariates and baseline instrument score, baseline trough FEV_1, and percent salbutamol reversibility as continuous covariates. Onset of action was assessed using a repeated measures ANCOVA fitted to Day 1 post-baseline data. The model included treatment, timepoint, prior maintenance medication (ICS *versus* ICS/LABA) and treatment-by-timepoint interaction as categorical covariates, and baseline trough FEV_1 and percent reversibility as continuous covariates. For rescue medication, a repeated measures ANCOVA model analysed change from baseline in average daily rescue salbutamol use. The model included treatment, the number of the relevant 4-week intervals (1 to 3), prior maintenance medication (ICS *versus* ICS/LABA), severe asthma exacerbation history (0, ≥1) and the treatment-by-4-week interval interaction as categorical covariates, and baseline daily

rescue salbutamol use, baseline trough FEV_1 and percent salbutamol reversibility as continuous covariates.

Supplementary Table S1. Summary of key inclusion and exclusion criteria.

Key inclusion criteria

- Aged 12–80 years (inclusive)
- A documented history of physician-diagnosed asthma ≥6 months before Visit 1
- Regularly using stable daily low-dose ICS or an ICS/LABA regimen ≥8 weeks before Visit 1
- A 7-item ACQ total score ≥1.5 at Visits 1 and 4
- A pre-bronchodilator/pre-dose FEV_1 <90% of predicted normal at Visits 1, 2 and 3, and a pre-dose FEV_1 of 50%–90% at Visit 4 (pre-randomisation)
- Exhibiting reversibility to salbutamol, defined as a post-salbutamol increase in FEV_1 of ≥12% and ≥200 mL in those ≥18 years or a post-salbutamol increase in FEV_1 of ≥12% in those 12 to <18 years of age, either in the 12 months before Visit 1 or at Visit 2 or Visit 3
- A pre-bronchodilator/pre-dose FEV_1 at Visits 2, 3 and 4 that has not changed by ≥20% (increase or decrease) from the pre-bronchodilator/pre-dose FEV_1 recorded at the previous visit
- Asthma stability during run-in based on symptom worsening assessments, including any of the following on at least 2 consecutive days: decreased morning peak expiratory flow of ≥20%, increased rescue salbutamol use (≥4 inhalations), increased nighttime awakenings (≥2 nights) or increased asthma symptoms

Key exclusion criteria

- Life-threatening asthma, defined as a history of significant asthma episode(s) requiring intubation associated with hypercapnia, respiratory arrest, hypoxic seizures or asthma-related syncopal episode(s)
- Hospitalisation for asthma within 8 weeks of Visit 1
- Completed treatment for any respiratory infection or asthma exacerbation treated with systemic corticosteroids and/or additional ICS treatment in the 8 weeks prior to Visit 1 and throughout screening
- Historical or current evidence of clinically significant disease including, but not limited to, cardiovascular, hepatic, renal, haematological, neurological, endocrine, gastrointestinal or pulmonary diseases
- Current smokers; former smokers with >10 pack-years history or who stopped smoking <6 months before Visit 1

- Participation in another clinical study with a study intervention administered in the last 30 days or 5 half-lives (whichever is longer)
- Participants with known hypersensitivity to β_2-agonists, corticosteroids or any component of the MDI
- Any clinically relevant abnormal findings in physical examination, clinical chemistry, haematology, vital signs or ECG

ACQ, Asthma Control Questionnaire; ECG; electrocardiogram; FEV_1, forced expiratory volume in 1 second; ICS, inhaled corticosteroids; LABA, long-acting β_2-agonist; MDI, metered-dose inhaler.

Supplementary Table S2. Summary of treatment differences for efficacy endpoints, (Europe/ROW regional approach), Efficacy Set.

	BFF_A 160	BD 160
**Primary endpoint**		
**Change from baseline in morning pre-dose trough FEV_1 over 12 weeks, mL**	n=177	n=169
Estimate (SE)	135 (17.4)	73 (17.8)
LS mean difference (95% CI), p-value	62 (13–111), 0.0133[a]	
**Secondary endpoints**		
**Change from baseline in FEV_1 $AUC_{0–3}$ over 12 weeks, mL**	n=177	n=169
Estimate (SE)	299 (17.3)	121 (17.7)
LS mean difference (95% CI), p-value	179 (130–227), <0.0001[a]	
**Onset of action: change in FEV_1 at 5 minutes post-dose on Day 1, mL**	n=168	n=157
Estimate (SE)	179 (13.8)	26 (14.2)
LS mean difference (95% CI), p-value	153 (114–192), <0.0001[a]	
**Percentage of 7-item ACQ responders over 12 weeks**	n=177	n=169
Responders, n (%)	127 (71.8)	107 (63.3)
Odds ratio (95% CI), p-value	1.512 (0.956–2.391), 0.0768	
**Percentage of 5-item ACQ responders over 12 weeks**	n=177	n=169
Responders, n (%)	134 (75.7)	116 (68.6)
Odds ratio (95% CI), p-value	1.465 (0.906–2.367), 0.1190	
**Percentage of AQLQ(s)+12 responders over 12 weeks**	n=174	n=163
Responders, n (%)	103 (59.2)	82 (50.3)
Odds ratio (95% CI), p-value	1.488 (0.933–2.374), 0.0951	
**Change from baseline in mean daily number of puffs of rescue medication use over 12 weeks (inhalations/day)**	n=171	n=167
Estimate (SE)	-0.589 (0.0708)	-0.309 (0.0721)
LS mean difference (95% CI),	–0.280 (−0.478, −0.082), 0.0058[a]	

p-value

[a]Significant per the Type I error control procedure.

ACQ, Asthma Control Questionnaire; AQLQ(s)+12, Asthma Quality of Life Questionnaire for 12 years and older; $AUC_{0–3}$, area under the curve over 0–3 hours post-dose; BD 160, budesonide 160 µg via Aerosphere® MDI; BFF_A 160, budesonide/formoterol fumarate dihydrate 160/10 µg via Aerosphere® MDI; CI, confidence interval; FEV_1, forced expiratory volume in 1 second; LS, least squares; MDI, metered-dose inhaler; ROW, rest of world.

Supplementary Table S3. Summary of the tertiary exploratory endpoint of symptom-free days over 12 weeks while on treatment, Efficacy Set

	**BFF_A 160**	**BD 160**
**Percentage of Symptom-free Days Over 12 Weeks: While on Treatment Estimand[a,b]**	n=171	n=167
**Estimate (SE)**	48.582 (1.8078)	43.035 (1.8435)
**LS mean difference (95% CI), p-value**	5.546 (0.477, 10.616), 0.0321[c]	

[a]Uses all observed data until the day prior to the occurrence of the first Intercurrent Event. [b]Assessed using a repeated measures ANCOVA model. The model included treatment, the number of the relevant 4-week interval (interval 1 to 3), prior maintenance medication (ICS vs ICS-LABA), severe asthma exacerbation history (0, ≥1) and the treatment-by-4-week interval interaction as categorical covariates, and baseline daily rescue salbutamol use, baseline trough FEV1, and percent salbutamol reversibility as continuous covariates. [c]Nominally significant due to absence of Type I error control.

BD 160, budesonide 160 μg via Aerosphere® MDI; BFF_A 160, budesonide/formoterol fumarate dihydrate 160/10 μg via Aerosphere® MDI; CI, confidence interval; FEV_1, forced expiratory volume in 1 second; LS, least squares; MDI, metered-dose inhaler; ROW, rest of world.

Supplementary Figure S1. Overview of Type I error control MTPs for A) US regional approach; B) EU/ROW regional approach.

A)

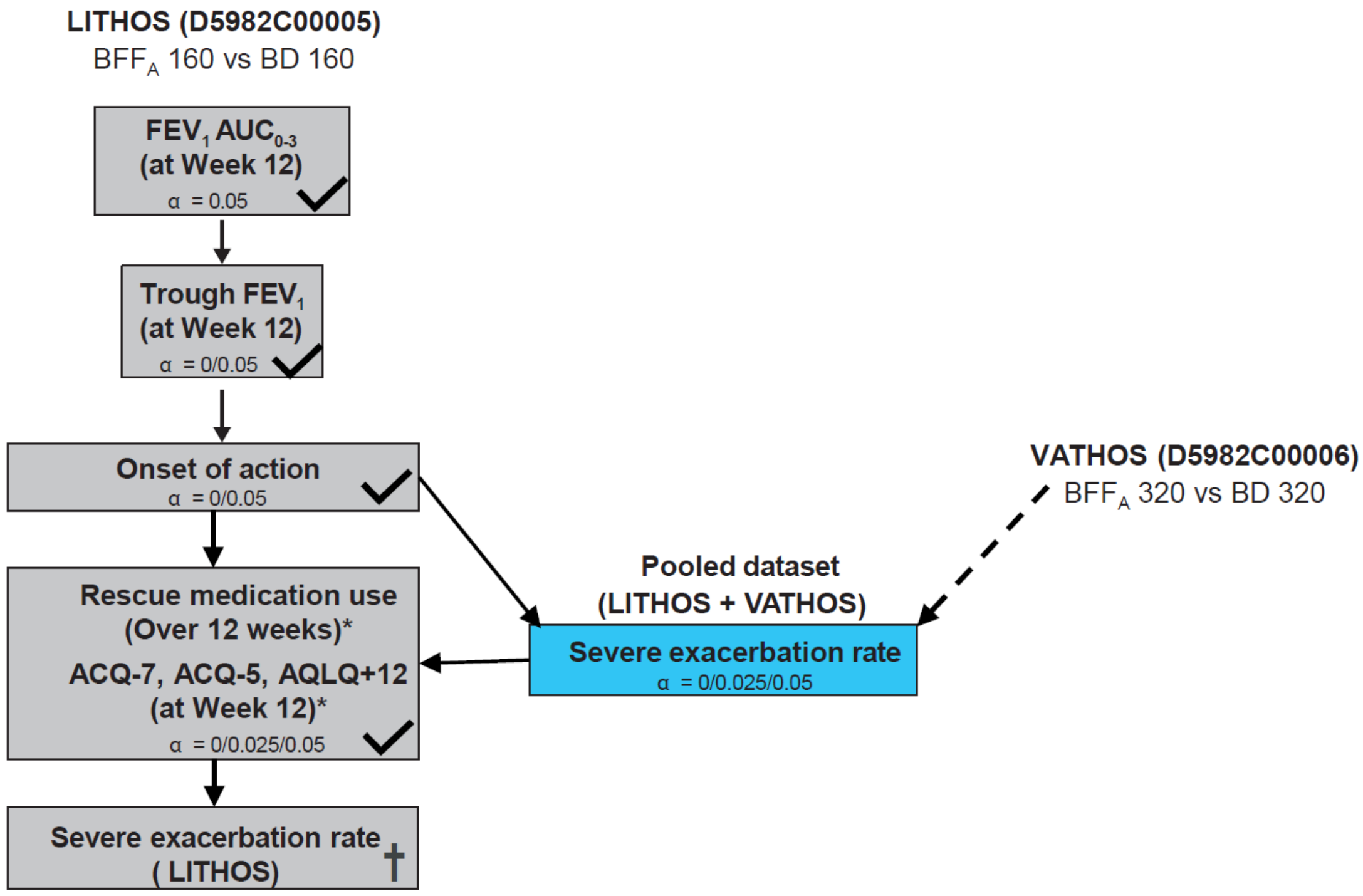

B)

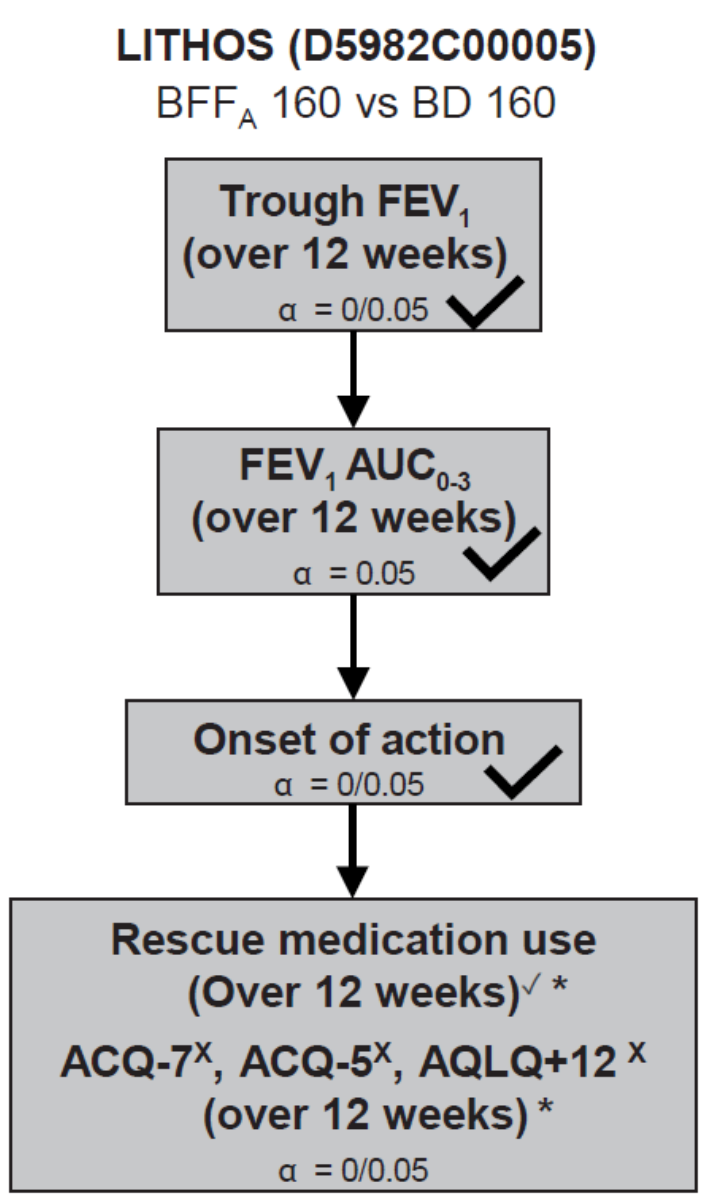

The endpoint based on the pooled dataset is reported in the VATHOS primary publication (NCT05202262).

*The test on change from baseline in puffs of rescue medication and the percentage responder endpoints for ACQ-7, ACQ-5, AQLQ-12 will be strongly controlled using a Hochberg procedure.

† denotes nominal significance due to placement in MTP; ✓ denotes statistical significance; X denotes no significance.

ACQ, Asthma Control Questionnaire; AQLQ(s)+12, Asthma Quality of Life Questionnaire for 12 years and older; $AUC_{0–3}$, area under the curve over 0–3 hours post-dose; BD 160, budesonide 160 µg via Aerosphere® MDI; BFF_A 160, budesonide/formoterol fumarate dihydrate 160/10 µg via Aerosphere® MDI; MTP, multiple testing procedure; ROW, rest of world.

Supplementary Figure S2. Subgroup analyses for primary endpoints of change from baseline in FEV_1 $AUC_{0–3}$ at Week 12 by US regional approach (A) and in morning pre-dose trough FEV_1 over 12 weeks by EU/ROW regional approach (B).

A

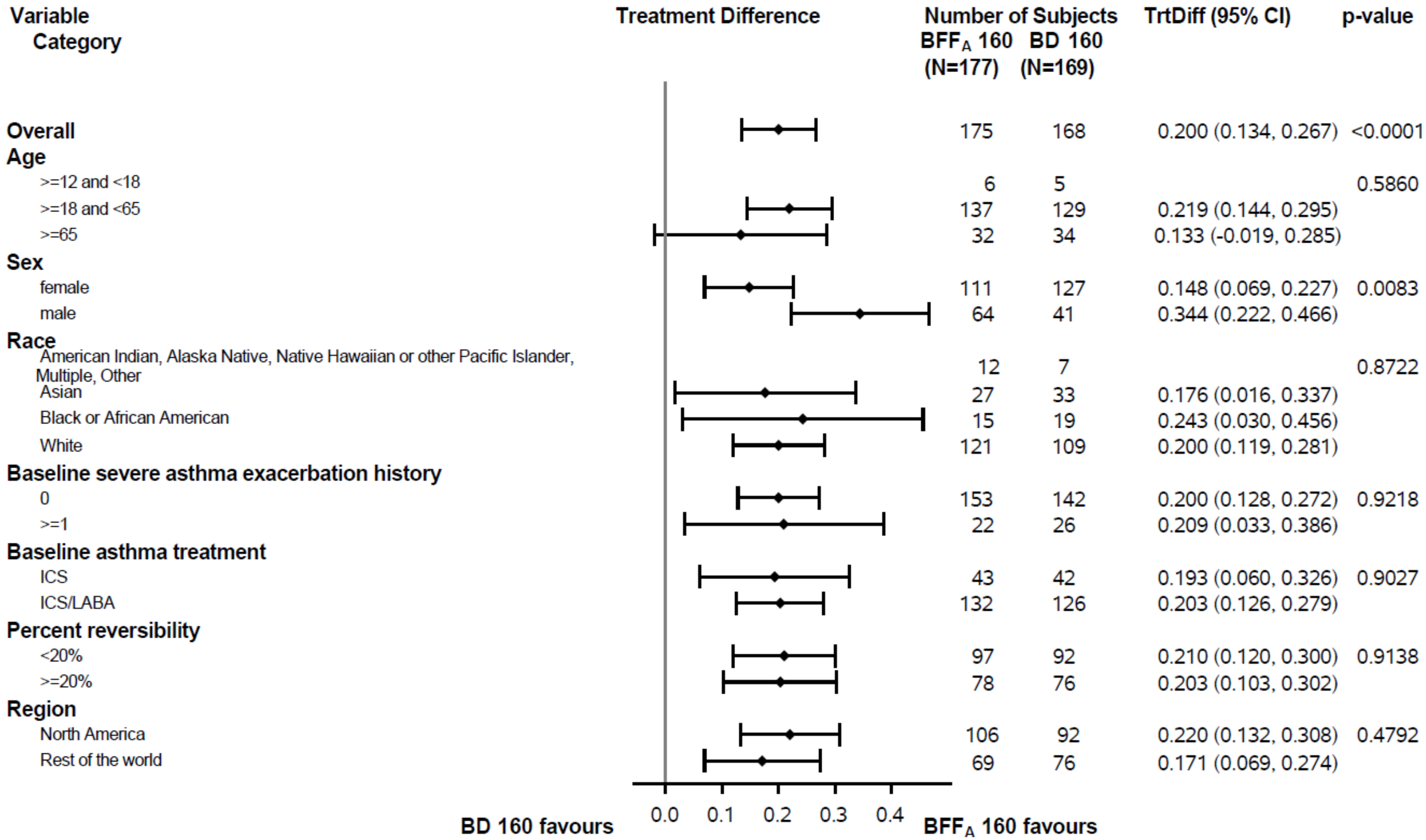

B

Variable / Category	Treatment Difference	Number of Subjects BFF$_A$ 160 (N=177)	BD160 (N=169)	TrtDiff (95% CI)	p-value
**Overall**		175	168	0.082 (0.020, 0.144)	0.0096
**Age**					
>=12 and <18		6	5		0.9432
>=18 and <65		139	130	0.066 (0.011, 0.122)	
>=65		32	34	0.045 (-0.069, 0.158)	
**Sex**					
female		113	128	0.062 (0.003, 0.121)	0.8016
male		64	41	0.076 (-0.016, 0.167)	
**Race**					
American Indian, Alaska Native, Native Hawaiian or other Pacific Islander, Multiple, Other		12	7		0.7875
Asian		27	33	0.059 (-0.060, 0.178)	
Black or African American		15	19	0.046 (-0.112, 0.204)	
White		123	110	0.067 (0.007, 0.127)	
**Baseline severe asthma exacerbation history**					
0		154	142	0.054 (0.000, 0.107)	0.3816
>=1		23	27	0.116 (-0.014, 0.247)	
**Baseline asthma treatment**					
ICS		43	42	0.076 (-0.023, 0.175)	0.7428
ICS/LABA		134	127	0.057 (0.001, 0.114)	
**Percent reversibility**					
<20%		97	92	0.092 (0.026, 0.158)	0.2474
>=20%		80	77	0.034 (-0.039, 0.107)	
**Region**					
North America		108	93	0.044 (-0.021, 0.109)	0.4089
Rest of the world		69	76	0.086 (0.010, 0.163)	

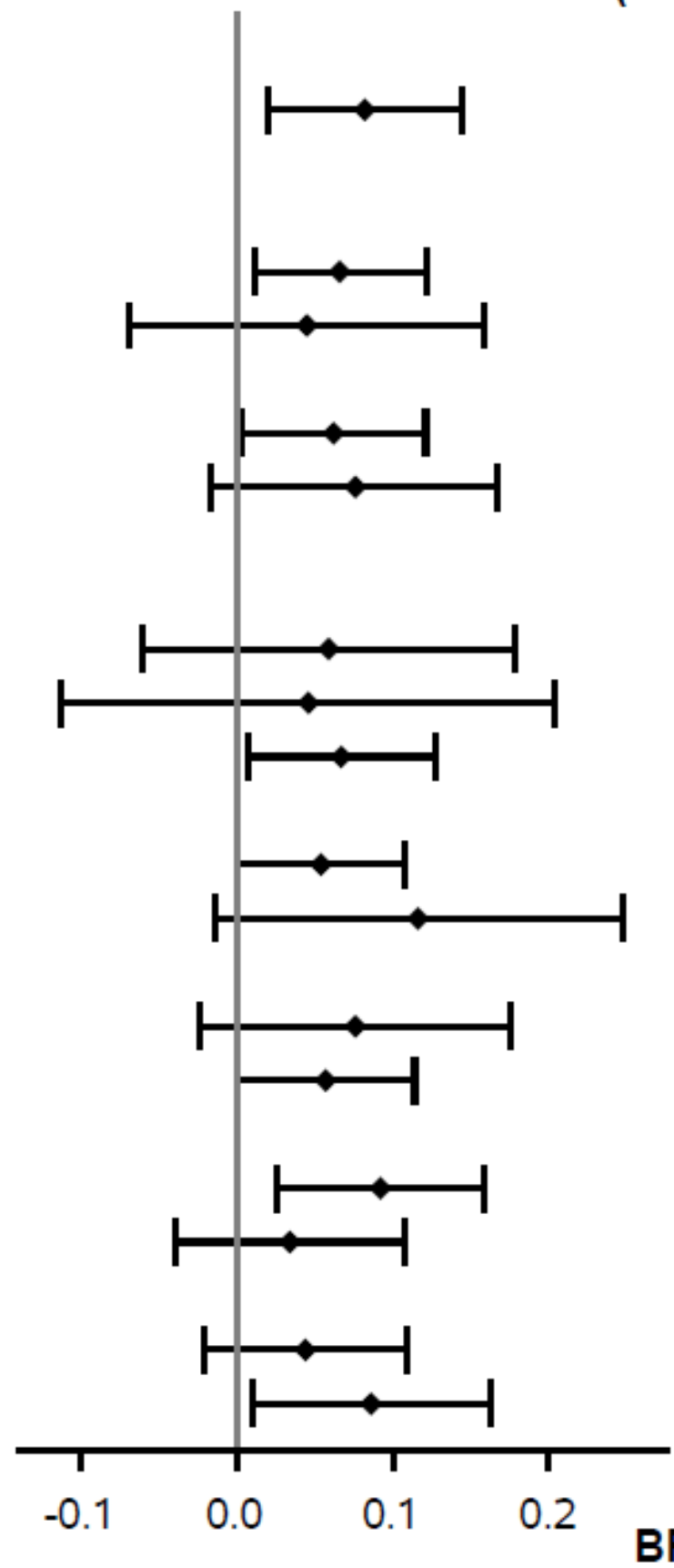

BD 160 favours **BFF$_A$ 160 favours**

The analysis was performed using a repeated measures analysis of covariance model. The model included treatment, subgroup, visit, prior maintenance medication (ICS versus ICS/LABA), treatment-by-visit interaction, treatment-by-subgroup interaction, subgroup-by-visit interaction, and treatment-by-subgroup-by-visit interaction as categorical covariates and baseline trough FEV_1 and percent salbutamol reversibility as continuous covariates.
For each subgroup analysis, if the subgroup parameter was same as covariate, the model factors were reduced by removing that covariate.
The interaction p-value refers to the difference in difference joint/F-test based on the subgroup interaction.
Baseline was defined as the average of the pre-dose FEV_1 measurements (at -60 and -30 minutes) at Visit 4 (Day 1).
Diamond denotes the point estimate.

$AUC_{0–3}$, area under the curve over 0–3 hours post-dose; BD 160, budesonide 160 µg via Aerosphere® MDI; BFF_A 160, budesonide/formoterol fumarate dihydrate 160/10 µg via Aerosphere® MDI; CI, confidence interval; FEV_1, forced expiratory volume in 1 second; ICS, inhaled corticosteroid; LABA, long-acting β_2-agonist; N, number of subjects per treatment group; ROW, rest of world; TrtDiff, treatment difference.

Supplementary Figure S3. Change from baseline in onset of action on Day 1, Efficacy Set.

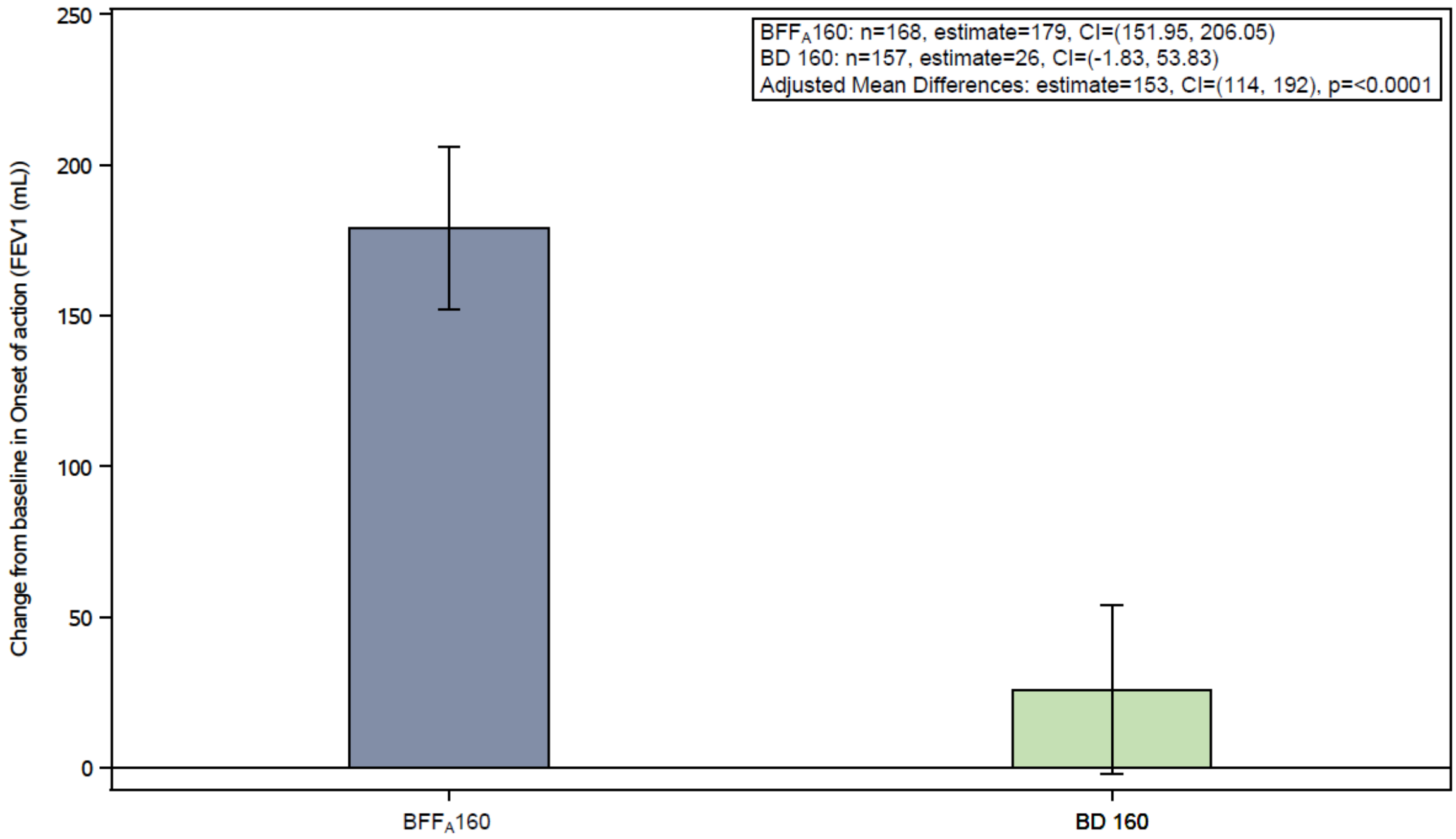

BD 160, budesonide 160 µg via Aerosphere® MDI; BFF_A 160, budesonide/formoterol fumarate dihydrate 160/10 µg via Aerosphere® MDI; CI, confidence interval; FEV_1, forced expiratory volume in one second; MDI, metered-dose inhaler.

Supplementary Figure S4. Change from baseline in ACQ-5 by timepoint

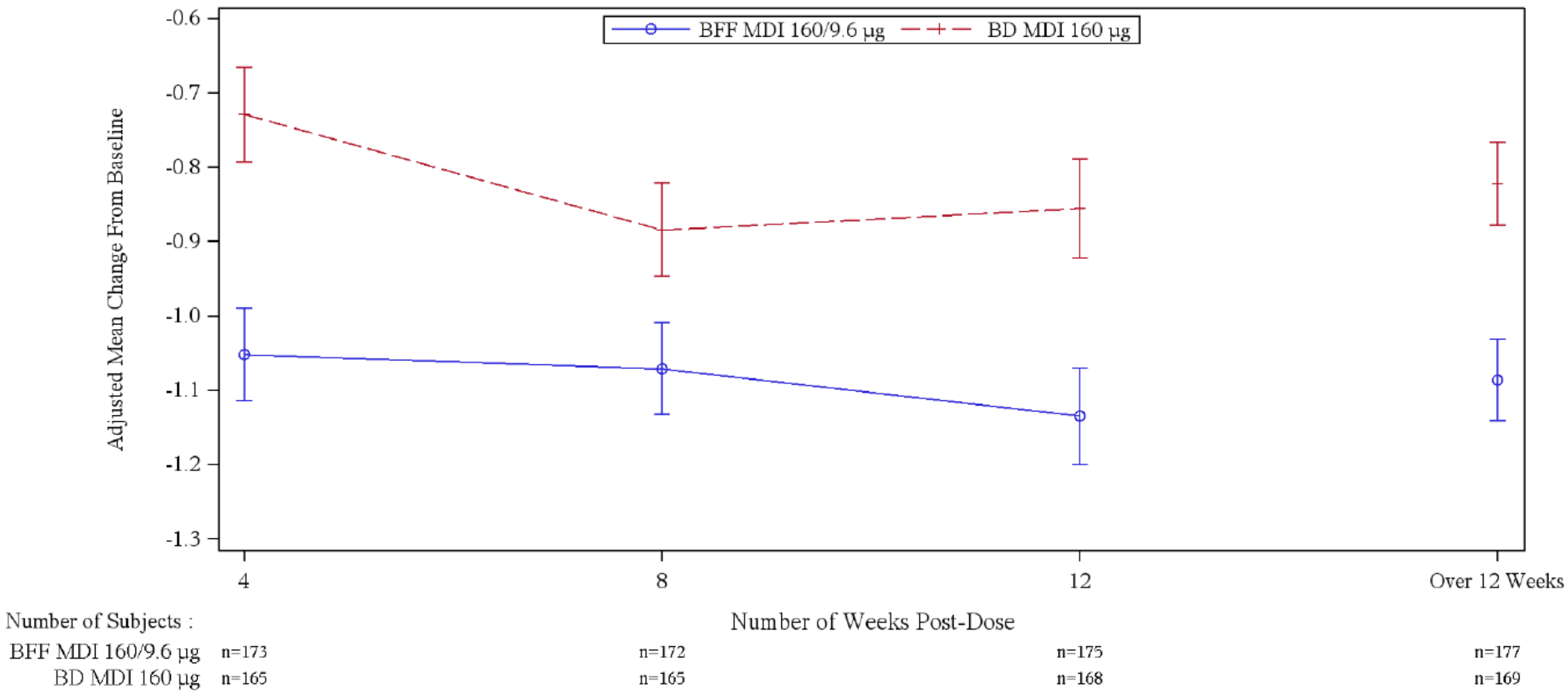

Data are the adjusted means and standard error from analysis of the efficacy dataset. Only subjects with non-missing covariates are included in the analysis. Baseline score for ACQ-5 was defined as the last non-missing ACQ-5 value before randomisation. The analysis was performed using a repeated measures analysis of covariance model. The model included treatment, visit, prior maintenance medication (ICS versus ICS/LABA), and the treatment-by-visit interaction as categorical covariates and baseline trough FEV_1, percent albuterol reversibility, and ACQ-5 baseline score as continuous covariates. Error bars show standard error of the mean (SE).
